# Association between high-dose methotrexate-induced toxicity and polymorphisms within methotrexate pathway genes in acute lymphoblastic leukemia

**DOI:** 10.3389/fphar.2022.1003812

**Published:** 2022-11-30

**Authors:** Meng Xu, Shuangshuang Wu, Yue Wang, Yundong Zhao, Ximin Wang, Changhong Wei, Xueying Liu, Feng Hao, Cheng Hu

**Affiliations:** ^1^ College of Laboratory Medicine, Jilin Medical University, Jilin, China; ^2^ School of Laboratory Medicine, Beihua University, Jilin, China; ^3^ Department of Pediatric Hematology, The First Hospital of Jilin University, Changchun, China; ^4^ National Engineering Laboratory for AIDS Vaccine, School of Life Sciences, Jilin University, Changchun, China; ^5^ Jilin Drug Inspection Center, Changchun, China; ^6^ Department of Hematology, The Linyi Central Hospital, Linyi, China

**Keywords:** acute lymphoblastic leukemia, high-dose methotrexate, toxicity, gene polymorphisms, mutation

## Abstract

Methotrexate (MTX) is a folic acid antagonist, the mechanism of action is to inhibit DNA synthesis, repair and cell proliferation by decreasing the activities of several folate-dependent enzymes. It is widely used as a chemotherapy drug for children and adults with malignant tumors. High-dose methotrexate (HD-MTX) is an effective treatment for extramedullary infiltration and systemic consolidation in children with acute lymphoblastic leukemia (ALL). However, significant toxicity results in most patients treated with HD-MTX, which limits its use. HD-MTX-induced toxicity is heterogeneous, and this heterogeneity may be related to gene polymorphisms in related enzymes of the MTX intracellular metabolic pathway. To gain a deeper understanding of the differences in toxicity induced by HD-MTX in individuals, the present review examines the correlation between HD-MTX-induced toxicity and the gene polymorphisms of related enzymes in the MTX metabolic pathway in ALL. In this review, we conclude that only the association of SLCO1B1 and ARID5B gene polymorphisms with plasma levels of MTX and MTX-related toxicity is clearly described. These results suggest that SLCO1B1 and ARID5B gene polymorphisms should be evaluated before HD-MTX treatment. In addition, considering factors such as age and race, the other exact predictor of MTX induced toxicity in ALL needs to be further determined.

## Introduction

Methotrexate (MTX) is widely used as a chemotherapy drug for malignant tumors. High-dose methotrexate (HD-MTX) is an effective treatment for extramedullary infiltration and systemic consolidation in children with acute lymphoblastic leukemia (ALL), and it can increase the long-term survival rate in children (Sajith et al., 2020). However, in patients treated with HD-MTX, it may lead to significant toxicity and may even interrupt cancer treatment, resulting in adverse anticancer effects (Widemann and Adamson, 2006). MTX-related toxicity depends on the dose, duration, genetic susceptibility, and risk factors (Mandal et al., 2020). There is heterogeneity associated with the pharmacokinetics and toxicity of the drug that is thought to be due to genetic differences in 68–75% of individuals (Radtke et al., 2013). These genetic differences may involve the activity of MTX metabolism-related enzymes, thus affecting the metabolism and induced toxicity of the drug.

Numerous clinical studies have focused on the pharmacogenetic factors affecting the pharmacokinetics of HD-MTX in childhood malignancies, so as to reasonably adjust the drug dose for the subsequent improvement of therapeutic effects and safety (Taylor et al., 2021). Mutations in key genes of the MTX metabolic pathway have been studied to determine their influence on individual differences (Radtke et al., 2013). These results suggest that genetic polymorphisms in metabolic enzymes associated with MTX may be potential predictors for personalized drug therapy. Herein, we systematically reviewed the research progress of gene polymorphisms of related enzymes in the MTX metabolic pathway affecting drug pharmacokinetics and toxicity. This revi**e**w aims to provide a basis for a more optimal understanding of the mechanism of MTX-induced toxicity in ALL.

### Application of methotrexate in acute lymphoblastic leukemia

ALL is the most common pediatric malignancy, accounts for around 80% of all leukemias and 30% of cancers in children (Pavlovic et al., 2019). The cumulative risk of developing leukemia in any child before the age of 15 is approximately 1:2,000, but this count masks the different age spectrum affected by each subtype. The incidence of ALL peaks in children aged 2–5 years and the disease has a B-cell precursor phenotype in the bone marrow (Greaves and Wiemels, 2003).

MTX is widely used in three distinct periods of ALL treatment in childhood including induction of remission, consolidation, and maintenance therapy (Kotur et al., 2020). Over the past few decades, improved treatment and administration regimens of MTX have led to significant increases in the survival rate of ALL patients (DeSantis et al., 2014). For a course of treatment, MTX is treated at doses that range from 12 mg intrathecally, 20 mg/m^2^/week orally to intramuscularly, or intravenously as high as 33,000 mg/m^2^/24 h for ALL (Jastaniah et al., 2018; Maksimovic et al., 2020).

Intravenous doses greater than or equal to 500 mg/m^2^ are defined as HD-MTX (Maksimovic et al., 2020). However, during treatment, 75% of patients will experience HD-MTX treatment-related side effects, and even 1–3% will die from drug-induced toxicity (Pavlovic et al., 2019). For example, HD-MTX crystallizes in the lumen of the renal tubules, and therefore, it can induce nephrotoxicity, leading to reduced clearance of MTX, and extended exposure to higher toxic concentrations can further degrade renal function and exacerbate adverse events, including hepatotoxicity, dermal toxicity, mucositis and bone marrow suppression (Howard et al., 2016). Therefore, the application of HD-MTX is hampered by the increased risk of dose concentration-induced toxicity, and treatment requires timely identification of delayed clearance of MTX and renal insufficiency.

Through routine observing of plasma MTX concentration and abnormal clearance, countermeasures such as timely rescue with leucovorin and enhanced hydration mechanisms can be taken to prevent excessive toxicity in the host (Holmboe et al., 2012). An effective indicator of MTX clearance and exposure during each treatment period is plasma MTX concentration, but it does not predict delayed excretion prior to MTX administration (Cheng et al., 2021). In addition, the pharmacokinetics, efficacy, and toxicity of MTX are heterogeneous. This diversity can be explained in part by differences in the sequence of genes encoding MTX metabolism (Moriyama et al., 2015). The current HD-MTX treatment regimens do not link the influence of genetic factors with drug clearance and exposure, but rather use body surface area (BSA)-based dosing. Therefore, it is essential to recognize gene polymorphisms of key enzymes of MTX metabolism before initiating HD-MTX treatment, as well as determine the appropriate dose of MTX and duration of leucovorin rescue to properly maximize efficacy while minimizing toxicity.

### Intracellular metabolic process of methotrexate

Folate is an extremely important enzyme cofactor mediating one-carbon unit transfer and is involved in several cellular biosynthetic pathways such as *de novo* synthesis of pyrimidines and purines, amino acid metabolism and mitochondrial protein synthesis (Wojtuszkiewicz et al., 2015). MTX is a folic acid antagonist that reduces DNA synthesis, repair, and subsequent cell proliferation by inhibiting several related enzymes in MTX metabolism involved in nucleotide biosynthesis, which ultimately leads to cell death (Abdi et al., 2021).

**FIGURE 1:**
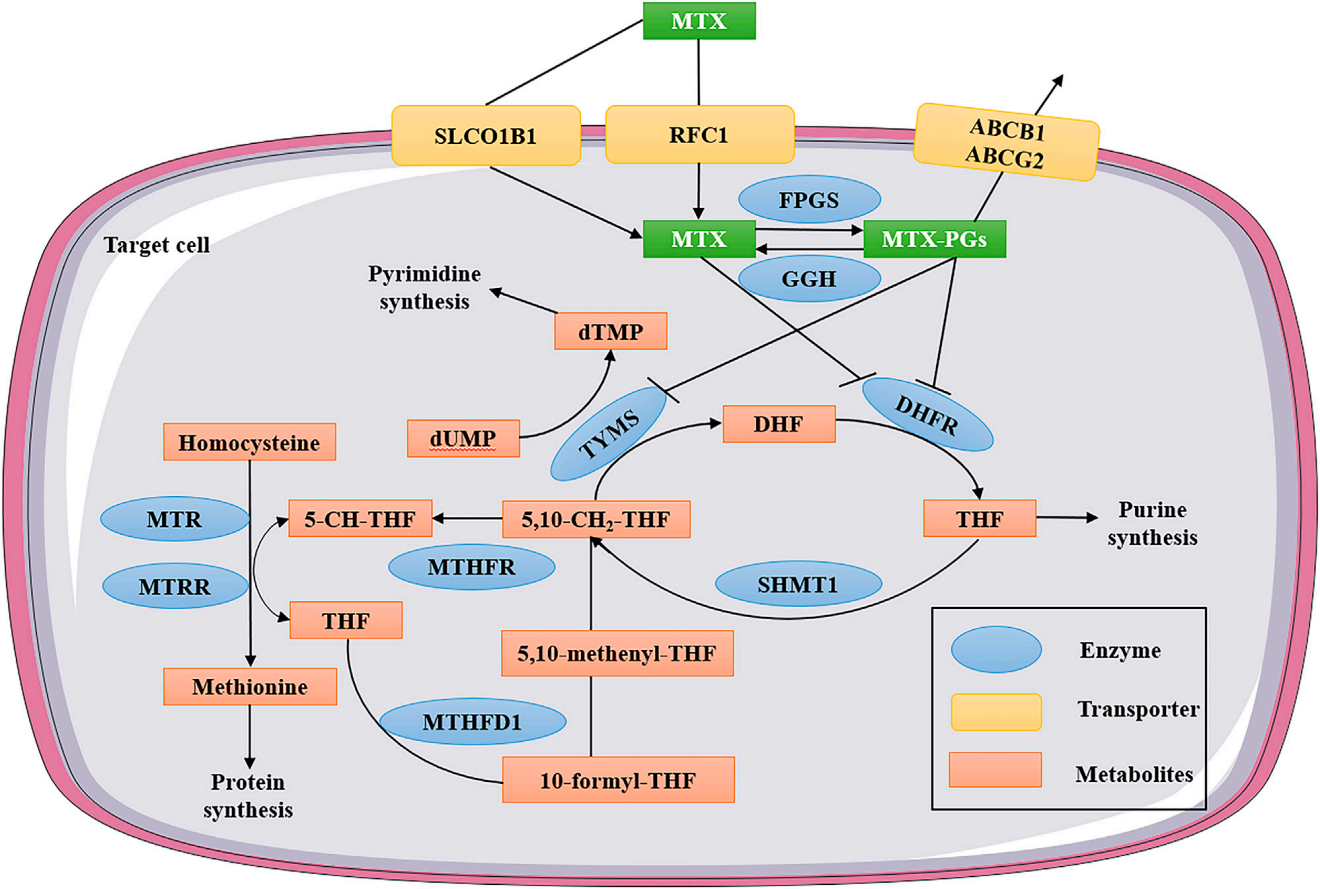
MTX intracellular folate metabolic pathway. MTX enters the cell through active transport mediated by RFC1 or SLCO1B1. After entering the cell, MTX is transformed into MTX-PGs under the action of FPGS. MTX-PGs is the sequential addition of multiple glutamate residues to the γ-carboxyl group of folate and MTX to ensure effective intracellular retention. At the same time, GGH converts MTX-PGs back to MTX. Subsequently, glutamate-free MTX was exported from the cell with the participation of members of the ATP-binding box superfamily (ABCB1 and ABCG2). The main targets of MTX and MTX-PGs are DHFR, TYMS and several other enzymes involved in purine synthesis. MTX—methotrexate; MTX-PGs—MTX polyglutamates; RFC1—reduced folate carrier 1; SLCO1B1-solute carrier organic anion transporter family member 1B1; ABCB1—ATP-binding cassette subfamily B member 1; ABCG2—ATP-binding cassette subfamily G member 2; FPGS—folylpolyglutamate synthetase; GGH—gamma-glutamyl hydrolase; DHFR—dihydrofolate reductase; TYMS—thymidylate synthetase; MTHFR—methylenetetrahydrofolate reductase; MTHFD1—methylenetetrahydrofolate dehydrogenase; MTR—methylenetetrahydrofolate-homocysteine methyltransferase; MTRR—5-methylenetetrahydrofolate-homocysteine methyltransferase reductase; SHMT1—serine hydroxymethyltransferase 1; THF—tetrahydrofolate; DHF—dihydrofolate; dUMP—deoxyuridine monophosphate; and dTMP—deoxythymidine monophosphate.

Active transport mediated by reduced folate carrier 1 (RFC1) or the solute carrier organic anion transporter 1B1 (SLCO1B1) allows MTX to enter the cell (see Figure1) (Liu et al., 2017), and when applying of HD-MTX therapy, passive diffusion also occurs to some extent through the cell membrane (Schmiegelow, 2009). Upon entry into the cell, MTX is transformed into MTX polyglutamates (MTX-PGs) under the action of folylpolyglutamate synthetase (FPGS) (Kato et al., 2012). MTX-PGs are based on the sequential addition of multiple glutamate residues to the gamma carboxyl groups of folic acid and MTX, ensuring effective intracellular retention and thus maintaining and enhancing target enzyme inhibition (Yang et al., 2017). Gamma-glutamyl hydrolase (GGH) converts MTX-PGs back to MTX, because it can remove glutamate from MTX-PGs (Chen and Shen, 2015). Subsequently, Glutamate-free MTX is exported from cells with the participation of the ATP-binding cassette superfamily such as ATP-binding cassette subfamily B member 1 (ABCB1) and ATP-binding cassette subfamily G member 2 (ABCG2) (Suthandiram et al., 2014). The main targets of MTX-PGs are dihydrofolate reductase (DHFR) and several other enzymes involved in purine synthesis (Chen and Shen, 2015). MTX and its polyglutamates can competitively inhibit DHFR, which depletes the tetrahydrofolate (THF) required by cells. THF is essential for DNA synthesis, and MTX disrupts the *de novo* biosynthesis of purines and pyrimidines by blocking tetrahydrofolate synthesis (Ghodke-Puranik et al., 2015).

5-methyl-THF, 10-formyl-THF and 5,10-Methyl-THF are several different forms of active folate present in organisms, which are donors of one-carbon units such as methylene, formyl and methyl in that order (Assaraf, 2006). MTX also inhibits other enzymes involved in the intracellular metabolic processes, including methylenetetrahydrofolate reductase (MTHFR), methylenetetrahydrofolate dehydrogenase (MTHFD1), 5-aminoimidazole-4-carboxamide ribonucleotide formyltransferase/IMP cyclohydrolase (ATIC), thymidylate synthase (TYMS), 5-methylenetetrahydrofolate-homocysteine methyltransferase (MTR), and 5-methylenetetrahydrofolate-homocysteine methyltransferase reductase (MTRR) (see Figure1) (Mikkelsen et al., 2011). Single nucleotide polymorphisms (SNPs) in folic acid metabolic pathways, transport molecules, and transcriptional protein genes have been extensively studied to determine their effects on the pharmacokinetics and HD-MTX induced toxicity in ALL patients (Cao et al., 2018; Giletti and Esperon, 2018). However, the previous pharmacological research results of MTX have been controversial partly due to confounding factors used in result analysis, such as differences in therapy protocols and study populations (Cheng et al., 2021). Next, we will systematically summarize the correlation between the polymorphisms of various metabolically related enzymes and MTX-induced toxicity.

## Gene polymorphisms of transporters

### The solute carrier family

The *RFC1* gene encodes the solute carrier family 19, member 1(SLC19A1) and is located on the long arm of chromosome 21 (Niedzielska et al., 2013). SLC19A1 is a widespread transporter that induces uptake of endogenous folate reduction and anti-folate exogenous substance including MTX for maintain folate homeostasis (Uhlén et al., 2015). SLC19A1 plays a crucial role in the transport of MTX into cells, and the *RFC1* 80G>A polymorphism (rs1051266) is a common SNPs occurring in the second exon of the *RFC1* gene (He et al., 2014). It results in the substitution of guanine for adenine at nucleotide 80, which in turn leads to the substitution of arginine for histidine at protein residue 27, resulting in reduced transport of anti-folate chemotherapeutic drugs (Gomez-Gomez et al., 2019).

However, whether the *RFC1 80G>A* mutation is associated with the risk of all remains controversial. A study by Chiusolo et al. found the *RFC1* 80G>A polymorphism is not related to the MTX plasma levels and MTX-induced toxicity, but the *RFC1* 80G>A polymorphism is significantly different in a better overall survival (OS) and progression-free survival (PFS) (Chiusolo et al., 2012). In another study, Gregers et al. found that among the three genetic polymorphisms 80AA, GG and GA in children treated with HD-MTX, patients with the 80AA genotype showed a higher level of myelotoxicity, but patients with the 80 GG genotype showed a higher level of hepatotoxicity (Gregers et al., 2010). In addition, a meta-analysis indicated that no association was discovered between the *RFC1* G80A polymorphism and ALL risk of disease, and the same is true for the sub-analysis by race (Forat-Yazdi et al., 2016).

In a study on the influence of *RFC1* SNPs and haplotypes on HD-MTX-induced toxicities in 88 adolescents and children with ALL, authors revealed that the rs2838958 TT genotype were more likely to develop mucositis in patients, compared to carriers with at least one rs2838958 C allele, and haplotype TGTTCCG (H4) notably decreased the risk of adverse events in response to HD-MTX therapy (Kotnik et al., 2017). Collectively, the limited sample size may be one reason for the inconsistencies in the studies published to date.

The *SLCO1B1* gene locates on chromosome 12 and encodes organic anion transporting polypeptide 1 (OATP1B1) (Ferrari et al., 2014). OATP1B1 is a genetically polymorphic influx transporter, that mainly expressed in the basolateral membrane of hepatocytes in the liver and plays an important role as a transporter protein for many endogenous compounds and drugs (Niemi et al., 2011).

A study conducted by Lopez-Lopez et al. showed that patients with the rs4149081 AA genotype and rs11045879 CC genotype were related to higher MTX plasma levels in 115 Spanish pediatric B-ALL patients receiving HD-MTX. Thus, this may be a reliable tool for detecting MTX clearance in low-risk patients to avoid MTX-related toxicity (Lopez-Lopez et al., 2011). In later studies, Li et al. analyzed a correlation between two polymorphisms (rs4149081 and rs11045879) in the *SLCO1B1* gene and MTX plasma concentration in 280 Chinese pediatric B-ALL patients, they also indicated that the rs11045879 CC genotype or the rs4149081 AA genotype was associated with high-MTX plasma concentrations (Li J. et al, 2015).

Besides, other *SLCO1B1* variants, such as the 521T>C polymorphism (SNP; rs4149056) was associated with HD-MTX clearance in childhood ALL in genome-wide association studies (GWASs), they found the clearance rate of the rs4149056 CC genotype was lower than that of the TT genotype (Ramsey et al., 2012; Ramsey et al., 2013). Further, a recent study also showed that the *SLCO1B1* 521T>C mutation reduced MTX clearance. The 521T>C genotype also acted on the 388A>G (SNP; rs2306283) variant and was associated with increased clearance of the 388G allele only when the 521T>C genotype was stratified (Schulte et al., 2021). Taken together, *SLCO1B1* variants were universally associated with MTX clearance, as differences MTX clearance has been significantly correlated with drug-induced toxicity and efficacy, especially the *SLCO1B1* rs11045879 CC genotype and rs4149081 AA genotype are associated with high MTX plasma concentrations and low MTX clearance. These results suggest that the *SLCO1B1* gene polymorphisms is a satisfactory genetic predictor of MTX plasma concentrations and MTX clearance.

### The ATP-binding cassette family

ABCB1 is known as a multidrug-resistant protein. The *ABCB1* gene is located on chromosome 7 and encodes for P-glycoprotein (P-gp), a membrane transporter that is a member of the ATP-binding cassette (ABC) superfamily (Juliano and Ling, 1976). ABCB1 is expressed in the liver, kidneys, and gastrointestinal tract (Uhlén et al., 2015). A meta-analysis indicated that there is statistical evidence that the ABCB1 polymorphism is related to an increased risk of ALL in Asians (Zhang et al., 2015). Zaruma-Torres et al. found that the *ABCB1* 1936A>G and 2107A>G polymorphisms were not associated with MTX adverse reactions (ADRs), but there was a protective effect for myelosuppression from the *ABCB1* 1236T>C (rs1128503) polymorphisms (Zaruma-Torres et al., 2015). A recent study by Mohammad indicated that higher concentrations of plasma MTX occurred in those with the *ABCB1* rs1045642 polymorphism CT genotype. They also showed that the *ABCB1* rs 1045642 CT and TT genotypes had a poorer event-free survival (EFS) than patients harboring CC genotype (Esmaili et al., 2020). However, in another study, Cheng et al. failed to find a correlation between MTX plasma levels and the *ABCB1* rs1045642 and rs1128503 polymorphic variants (Cheng et al., 2021).

ABCG2 protein is encoded by ABCG2 gene. It is second member of the ABC transporter G subfamily, which is known as breast cancer resistance protein (BCRP), and overexpression of ABCG2 in tumor cell lines *in vitro* has been shown to induce resistance to several anticancer drugs, including MTX (Wang et al., 2011).It is a transmembrane protein made up of 665 amino acids (Robey et al., 2009). ABCG2 protein is broadly expressed in the gastrointestinal tract, liver, kidney, and brain endothelium and play an important role in efflux, including the excretion of xenobiotics such as chemotherapeutic agents from cells (Zhang et al., 2018). Mesallamy et al. examined the effect of the *ABCG2* 421C>A (rs2231142) mutation on plasma levels of MTX in Egyptian pediatric ALL patients, and they showed that there was no correlation between the *ABCG2* 421C>A and MTX plasma levels (El Mesallamy et al., 2014). However, another study reported that the *ABCG2* 421C>A genotype is associated with higher MTX plasma levels (Esmaili et al., 2020). Based on all of the above studies, these inconsistent results may be due to different sample sizes and SNP selection criteria.

## Gene polymorphisms in the folate pathway

### Folylpolyglutamate synthetase and gamma-glutamyl hydrolase

It is widely known that the pharmacological activity of MTX is determined by its intracellular concentration and retention time (Masson et al., 1996). Some studies have exhibited that the activities of FPGS and GGH real-time determine the intracellular concentration of MTX (Wang et al., 2014). First, MTX is rapidly converted to MTX-PGs by FPGS, and GGH then turns long-chain MTX-PGs into short-chain MTX-PGs by removing polyglutamates and eventually into MTX, which is released from the cells into the bloodstream (van der Straaten et al., 2007). Liu et al. found that the FPGS rs1544105 polymorphism may affect the treatment outcome of BCP-ALL patients by influencing the expression of FPGS, but the rs1544105 polymorphism was not shown to be associated with any MTX-induced toxicity (Liu et al., 2013). In another study, the authors reported that the MTX plasma concentration of patients with the FPGS rs1544105 AA was considerably higher than that of patients with the FPGS rs1544105 GA or GG after 24 h of MTX treatment, but no differences were found after 44 h (Huang et al., 2016). Moreover, the concentration of MTX in the FPGS rs1544105 GG carriers was lower than that in GA or AA carriers, and the concentration of MTX in the GGH rs3758149 CC carriers was considerably lower than that in CT or TT carriers (Wang et al., 2014). Given all of that, these data suggested that the FPGS and GGH polymorphisms are associated with MTX plasma concentrations and that detecting their genotype may guide the dosage of MTX to reduce MTX-induced toxicity.

### Dihydrofolate reductase

DHFR is a key enzyme in the midst of thymidylate synthesis. It catalyzes the reduction of folic acid to dihydrofolate (DHF) and also converts DHF to tetrahydrofolate (THF) (Neradil et al., 2012). The major mechanism of MTX action is the suppression of dihydrofolate reductase encoded by the *DHFR* gene (Jekic et al., 2016). However, only a few ALL studies have examined the relationships between DHFR and MTX-induced adverse effects. Alessia et al. reported that homozygosity of the 19-bp deleted allele of DHFR significantly increased hepatotoxicity in adult ALL (Ongaro et al., 2009). Ceppi et al. analyzed the genotypes of six tag polymorphisms and the resulting haplotypes for a link with ALL outcome. They found that the DHFR SNPs A-680C, A-317G and C-35T with the lowest EFS in childhood ALL high-risk patients, and there was a remarkably higher risk of events for carriers of haplotype *1 compared with noncarriers and a lower likelihood of EFS (Ceppi et al., 2018).

In another study, the authors revealed that the intergenic SNP rs1382539 located in the DHFR regulatory element was related to increased levels of short-chain MTX-PGs due to inhibition of enhancer activity (Tulstrup et al., 2020). Collectively, the DHFR polymorphisms may be associated with reduced EFS and increased MTX plasma levels, but no consistent results have supported the association of the DHFR polymorphism with MTX-induced toxicity.

### Thymidylate synthase

TYMS is an enzyme participated in the *de novo* biosynthesis of purines and deoxythymidylate, that the enzyme is directly inhibited by MTX-PGs (Fotoohi et al., 2004). It catalyzes dUMP into dTMP and 5,10-methylene-THF into DHF(Muralidharan et al., 2017). Three common polymorphisms in the *TYMS* gene have been reported, that including different repetitions of a 28-nucleotide region (rs34743033; 2R3R), a G>C exchange in the 12th nucleotide in the second repetition of the 3R allele, and a 6-bp deletion in the 3' untranslated region (UTR) (3'UTR 6-bp ins/del, rs16430) (Lincz et al., 2007). In a study that enrolled 117 pediatric patients with ALL, the researchers found that the *TYMS* 6-bp deletion and 2R3R polymorphism were not related to MTX-induced oral mucositis (Oosterom et al., 2018).

In addition, a recent study reported an increased risk of vomiting for those with the 2R3R genotype of the *TYMS* gene and vomiting and hepatotoxicity for those with the homozygous 3R allele (Cwiklinska et al., 2020). These results regarding the effect of *TYMS* gene polymorphisms on producing different MTX-induced toxicity are controversial.

### Methylenetetrahydrofolate reductase

MTHFR is an enzyme that irreversible converts 5,10-CH_2_-THF, which is essential for purine and thymidine synthesis, into 5-CH-THF, which is essential for protein synthesis and nucleic acid methylation (Lopez-Lopez et al., 2013). The changes of MTHFR activity can modify folate pools *in vivo*, and then alter the response of normal and malignant cells on MTX-induced toxicity (De Mattia et al., 2009). Due to MTHFR plays a critical role in intracellular folate homeostasis and metabolism, there has been considerable research on MTHFR. The *MTHFR* gene is located on 1p36.3, and its most common SNP are the C677T (rs1801133) and the A1298C (rs1801131), the former polymorphism results in the substitution of alanine by valine and the latter polymorphism results in the substitution of glutamate by alanine (Jokic et al., 2011). Both mutations inhibited MTHFR activity but the A1298C to a lesser degree than the C677T polymorphism (Chen et al., 2002).Zhao et al. revealed that the MTHFR C677T polymorphism did not affect MTX plasma levels, but was positively correlated with the risk of MTX-induced all grades and severe liver and gastrointestinal toxicity in Caucasians (Zhao et al., 2016). Another study by Han et al. showed that with respect to the MTHFR A1298C, there was a significantly lower frequency of hematopoietic toxicity in patients with CC and AC genotypes as compared to patients with the AA genotype. There was a significantly higher risk of hematopoietic toxicity for patients with the MTHFR rs1801133T allele as compared with those with the CC genotype (Han et al., 2021).

However, Erčulj et al. indicated that the MTHFR polymorphisms were not associated with HD-MTX-induced toxicity, but MTHFR 677C>T-TYMS 2R3R and MTHFD1 1958G>A-MTHFR 677C>T interactions were remarkablely associated with decreased HD-MTX-related toxicity (Erculj et al., 2012). In addition, a meta-analysis of the *MTHFR* C677T and A1298C polymorphisms was conducted for Asian, Caucasian, pediatric, and adult patients. The MTHFR C677T allele increased the odds of myelosuppression, oral mucositis, gastrointestinal, skin toxicity, and hepatotoxicity, and the *MTHFR* A1298C mutation may be related to reduced risk of skin toxicity and leukopenia (Campbell et al., 2016). Together, these data indicated that the MTHFR C677T polymorphism may be positively associated with the risk of MTX-related severe toxicity and the MTHFR A1298C polymorphism may be associated with reduced risk of MTX-related toxicity.

### Methylenetetrahydrofolate dehydrogenase

The trifunctional enzyme MTHFD1 catalyzes THF reduction, which results in analogous 10-formyl, 5,10-methenyl, and 5,10-methylene derivatives, which are important cofactors for methionine, thymidylate, and *de novo* purine synthesis (Krajinovic, 2008). The common SNP is the 1958G>A (rs2236225) polymorphism, which leads to conserved amino acid substitution with Arg653Gln in the 10-formyl-THF synthetase domain of the MTHFD1 complex and thus decreased enzyme activity (Christensen et al., 2009). Erčulj et al. found that the MTHFD1 1958A allele notably decreased the odds of hepatotoxicity in 167 ALL children (Erculj et al., 2012). Furthermore, Csordas et al. analyzed 63 SNPs of 14 genes and showed that the *SLC19A1, MTR* and *MTHFD1* gene variants were proven to be related to the progression of acute toxicity after HD-MTX treatment, among them the MTHFD1 rs2236225 showed potential for reduced hepatotoxicity (Csordas et al., 2014).

### Methyltransferase and 5-methylenetetrahydrofolate-homocysteine methyltransferase reductase

MTR catalyzes the methylation of homocysteine to methionine, which uses 5-methyl-THF as a methyl donor and cobalamin (vitamin B12) as a cofactor. After a period of time the cobalamin (I) cofactor of MTR is oxidized to form cobalamin (II), leading to inactivation of MTR, and MTRR oxidized cobalamin (II) to CH3-cobalamin (III) to restore MTR activity by reductive methylation (Li W. X. et al, 2015). The MTR gene is positioned at 1q43. The MTR A2756G (rs1805087) polymorphism causes the substitution from aspartic acid to glycine at codon 919 (D919G) and may change enzymatic activity (Harmon et al., 1999; Matsuo et al., 2002). Nikbakht et al. found that there were no significant differences for the MTR A2756G genotypes or the MTHFR (C677T and A1298C) polymorphisms and their interaction between patients and controls. No correlations between SNPs of the *MTR* gene and risk of ALL were observed (Nikbakht et al., 2012). A review performed to determine whether the MTR A2756G polymorphisms can individually reduce the risk of childhood ALL remained inconclusive, and further investigation is required (Li et al., 2014).

The *MTRR* gene A66G polymorphism (5p15.31a, rs1801394) leads to the substitution of isoleucine with methionine at codon 22 (Leclerc et al., 1998; Wilson et al., 1999).The MTRR polymorphisms have been less studied (Han et al., 2012). It should be noted that Kotnik et al. did not find an association between MTX-related toxicity and the genotype MTRR 66A>G (Faganel Kotnik et al., 2011). The analysis from another study indicated that the MTRR rs1801394 (A66G) and rs1532268 (C524T) genetic polymorphisms were not associated with acute leukemia (Amigou et al., 2012). However, one study showed that the rs1801394 (AG and GG) variants were related to lower MTX concentration at 24 h as compared to AA wild types (den Hoed et al., 2015).

### ATIC

Aminoimidazole carboxamide adenosine ribonucleotide (AICAR) transformylase (ATIC) is the enzyme that converts AICAR into formyl-AICAR (FAICAR) in the *de novo* purine synthesis pathway. It can be directly inhibited by MTX-PGs, leading to intracellular accumulation of AICAR (Dervieux et al., 2004). Park et al. assessed correlations between the ATIC 347C>G polymorphism and HD-MTX-induced toxicities. They reported that there were no correlations between the 347C>G polymorphism of ATIC and HD-MTX-induced adverse events (Park and Shin, 2016). Additionally, no significance for ATIC was found in the GWAS related to MTX clearance (Ramsey et al., 2012). These results suggested that no correlations between the ATIC 347C>G polymorphism and MTX-induced toxicity and MTX clearance.

### Gene polymorphisms in cell development

The AT-rich interactive domain (ARID) 5 B (*ARID5B*) gene encodes one of the transcription factors ARID family that play an important role in embryonic development and growth regulation (Gutierrez-Camino et al., 2013). It is reported that the *ARID5B* gene is a novel susceptibility factor for pediatric B-cell ALL (Healy et al., 2010). As a result, it has been shown that some ARID5B SNPs were related to increased susceptibility to ALL in females as compared to males and *vice versa*. And that, there was a higher risk for pediatric ALL with the *ARID5B* haplotype block CGAACACAA (Al-Absi et al., 2017).

In addition, Wang et al. indicated that the ARID5B SNPs were associated with higher MTX-PGs levels in B lymphocytes, which provides a possible explanation for the stronger response of B-ALL to MTX (Wang et al., 2020).Csordas et al. found that the ARID5B rs4948502, rs4948496, and rs4948487 were associated with MTX serum levels and there were significant associations between hepatotoxicity and the ARID5B rs4948502 in patients who received 5 g/m^2^ MTX (Csordas et al., 2014). Further, a meta-analysis indicated that the *ARID5B* variants rs10821936, rs10994982, and rs7089424 were significantly related to the increased risk of childhood ALL (Yang et al., 2019). Collectively, the *ARID5B* variants were related to MTX serum levels and the increased risk of childhood ALL. Further studies with a larger sample size and functional studies are required to ascertain the variants of the *ARID5B* gene polymorphism and their impacts on increased ALL risk or toxicity.

## Conclusion

In this review, we focused on the relevance of MTX-related metabolic gene polymorphisms (SLC family, ABC transporter, FPGS, GGH, DHFR, TYMS, MTHFR, MTHFD1, MTR, MTRR, ATIC, and ARID5B) to MTX plasma levels and MTX-induced toxicity (Table 1). Thus far, it is universally accepted that the SLCO1B1 polymorphisms are associated with MTX-related toxicity and increased clearance as well as decreased clearance. The *ARID5B* variants were significantly associated with an increased risk of pediatric ALL. Therefore, the association of *SLCO1B1* and *ARID5B* gene polymorphisms with plasma levels of MTX and MTX-related toxicity is important and should probably be evaluated prior to HD-MTX therapy.

**TABLE 1:** Gene polymorphisms and their correlation with MTX plasma levels and MTX-induced toxicities.

SNP	Variant (genotype)	Toxicity	MTX plasma concentration	MTX clearance	References
*RFC1* rs1051266	G>A	—	—		Chiusolo et al., (2012); Forat-Yazdi et al., (2016)
	AA	↑ Bone marrow toxicity			Gregers et al. (2010)
	GG	↑ Liver toxicity			Gregers et al. (2010)
*RFC1* rs2838958	TT	↑ Mucositis			Kotnik et al. (2017)
*SLCO1B1* rs11045879	CC	↑	↑	↓	Lopez-Lopez et al., (2011); Li J et al., (2015)
*SLCO1B1* rs4149081	AA	↑	↑	↓	Lopez-Lopez et al., (2011); Li J et al., (2015)
*SLCO1B1* rs4149056	T>C	—		↓	Ramsey et al., 2012; Ramsey et al., 2013; Schulte et al., 2021
*SLCO1B1* rs2306283	A>G	—		↑	Schulte et al. (2021)
*ABCB1* rs1128503	T>C	↓Myelosuppression			Zaruma-Torres et al., (2015); Wojtuszkiewicz et al., (2015)
*ABCB1* rs1045642	C>T	—	↑/—		Esmaili et al. (2020); Wojtuszkiewicz et al., (2015)
	CT	—			Esmaili et al. (2020)
	TT	—			Esmaili et al. (2020)
ABCG2 rs2231142	C>A	—	↑/—		Esmaili et al. (2020); El Mesallamy et al., (2014)
*FPGS* rs1544105	G>A	—			Liu et al. (2013)
	AA	—	↑		Huang et al. (2016)
	GG	—	↓		Wang et al. (2014)
GGH rs3758149	CC	—	↓		Wang et al. (2014)
*MTHFR* rs1801133	C>T	↑ Hepatic and gastrointestinal toxicities/—			Zhao et al., (2016); Erculj et al., (2012); Campbell et al., (2016)
	TT	↑ Hematopoietic toxicity			Esmaili et al., (2020); Han et al., (2021)
*MTHFR* rs1801131	A>C	↓ Skin toxicity			Campbell et al., (2016)
	AC	↓Hematopoietic toxicity			Chiusolo et al., (2012); Campbell et al., (2016)
	CC	↓ Hepatotoxicity			Chiusolo et al., (2012);
*MTHFD1* rs2236225	G>A	↓Hepatotoxicity			Erculj et al., (2012); Csordas et al., (2014)
*ARID5B* rs4948502	C>T	↑	↑		Csordas et al., (2014); Wang et al., (2020)
		Hepatotoxicity			
↑, increase; ↓, decrease; —, no correlation.					

Although there have been numerous studies on the relationship between individual differences in MTX toxicity caused by changes in SNPs in these genes, unfortunately, no clear predictors have been identified. The reasons for this disappointing result include small or nonhomogeneous sample size; patient age, gender, and race; the use of non-objective toxicity markers; and different treatment options such as MTX dose and rescue time. The effect of other genetic and biochemical factors should be also considered. Gene-gene and gene-environment interactions will also influence the clinical outcome. The use of HD-MTX with other chemotherapeutic agents (doxorubicin, cyclophosphamide, and cytarabine) may also cause the MTX-related toxicities in patients. Therefore, considering race, age, and other factors, future work will focus on identifying the exact predictors of HD-MTX-induced toxicity in ALL.

## References

[B1] Abdi F. Mohammadi S. S. Falavarjani K. G. (2021). Intravitreal methotrexate. J. Ophthalmic Vis. Res. 16 (4), 657–669. 10.18502/jovr.v16i4.9756 34840688 PMC8593537

[B2] Al-Absi B. Noor S. M. Saif-Ali R. Salem S. D. Ahmed R. H. Razif M. F. (2017). Association of ARID5B gene variants with acute lymphoblastic leukemia in Yemeni children. Tumour Biol. 39 (4), 1010428317697573. 10.1177/1010428317697573 28381164

[B3] Amigou A. Rudant J. Orsi L. Goujon-Bellec S. Leverger G. Baruchel A. (2012). Folic acid supplementation, MTHFR and MTRR polymorphisms, and the risk of childhood leukemia: The ESCALE study (SFCE). Cancer Causes Control 23 (8), 1265–1277. 10.1007/s10552-012-0004-0 22706675

[B4] Assaraf Y. G. (2006). The role of multidrug resistance efflux transporters in antifolate resistance and folate homeostasis. Drug resist. updat. 9 (4-5), 227–246. 10.1016/j.drup.2006.09.001 17092765

[B5] Campbell J. M. Bateman E. Stephenson M. D. Bowen J. M. Keefe D. M. Peters M. D. (2016). Methotrexate-induced toxicity pharmacogenetics: An umbrella review of systematic reviews and meta-analyses. Cancer Chemother. Pharmacol. 78 (1), 27–39. 10.1007/s00280-016-3043-5 27142726

[B6] Cao M. Guo M. Wu D. Meng L. (2018). Pharmacogenomics of methotrexate: Current status and future outlook. Curr. Drug Metab. 19 (14), 1182–1187. 10.2174/1389200219666171227201047 29283070

[B7] Ceppi F. Gagné V. Douyon L. Quintin C. J. Colombini A. Parasole R. (2018). DNA variants in DHFR gene and response to treatment in children with childhood B ALL: Revisited in AIEOP-BFM protocol. Pharmacogenomics 19 (2), 105–112. 10.2217/pgs-2017-0153 29210328

[B8] Chen J. Ma J. Stampfer M. J. Palomeque C. Selhub J. Hunter D. J. (2002). Linkage disequilibrium between the 677C>T and 1298A>C polymorphisms in human methylenetetrahydrofolate reductase gene and their contributions to risk of colorectal cancer. Pharmacogenetics 12 (4), 339–342. 10.1097/00008571-200206000-00011 12042673

[B9] Chen Y. Shen Z. (2015). Gene polymorphisms in the folate metabolism and their association with MTX-related adverse events in the treatment of ALL. Tumour Biol. 36 (7), 4913–4921. 10.1007/s13277-015-3602-0 26022160

[B10] Cheng Y. Chen M. H. Zhuang Q. Lin B. J. Chen Y. Y. Yang L. (2021). Genetic factors involved in delayed methotrexate elimination in children with acute lymphoblastic leukemia. Pediatr. Blood Cancer 68 (5), e28858. 10.1002/pbc.28858 33501733

[B11] Chiusolo P. Giammarco S. Bellesi S. Metafuni E. Piccirillo N. De Ritis D. (2012). The role of MTHFR and RFC1 polymorphisms on toxicity and outcome of adult patients with hematological malignancies treated with high-dose methotrexate followed by leucovorin rescue. Cancer Chemother. Pharmacol. 69 (3), 691–696. 10.1007/s00280-011-1751-4 21984221

[B12] Christensen K. E. Rohlicek C. V. Andelfinger G. U. Michaud J. Bigras J. L. Richter A. (2009). The MTHFD1 p.Arg653Gln variant alters enzyme function and increases risk for congenital heart defects. Hum. Mutat. 30 (2), 212–220. 10.1002/humu.20830 18767138

[B13] Csordas K. Lautner-Csorba O. Semsei A. F. Harnos A. Hegyi M. Erdelyi D. J. (2014). Associations of novel genetic variations in the folate-related and ARID5B genes with the pharmacokinetics and toxicity of high-dose methotrexate in paediatric acute lymphoblastic leukaemia. Br. J. Haematol. 166 (3), 410–420. 10.1111/bjh.12886 24712521

[B14] Cwiklinska M. Czogala M. Kwiecinska K. Madetko-Talowska A. Szafarz M. Pawinska K. (2020). Polymorphisms of SLC19A1 80 G>A, MTHFR 677 C>T, and tandem TS repeats influence pharmacokinetics, acute liver toxicity, and vomiting in children with acute lymphoblastic leukemia treated with high doses of methotrexate. Front. Pediatr. 8, 307. 10.3389/fped.2020.00307 32612964 PMC7308427

[B15] De Mattia E. Toffoli G., (2009). C677T and A1298C MTHFR polymorphisms, a challenge for antifolate and fluoropyrimidine-based therapy personalisation. Pharmacogenomics J. 45 (8), 1333–1351. 10.1016/j.ejca.2008.12.004 19144510

[B16] den Hoed M. A. Lopez-Lopez E. te Winkel M. L. Tissing W. de Rooij J. D. Gutierrez-Camino A. (2015). Genetic and metabolic determinants of methotrexate-induced mucositis in pediatric acute lymphoblastic leukemia. Pharmacogenomics J. 15 (3), 248–254. 10.1038/tpj.2014.63 25348617

[B17] Dervieux T. Furst D. Lein D. O. Capps R. Smith K. Walsh M. (2004). Polyglutamation of methotrexate with common polymorphisms in reduced folate carrier, aminoimidazole carboxamide ribonucleotide transformylase, and thymidylate synthase are associated with methotrexate effects in rheumatoid arthritis. Arthritis Rheum. 50 (9), 2766–2774. 10.1002/art.20460 15457444

[B18] DeSantis C. E. Lin C. C. Mariotto A. B. Siegel R. L. Stein K. D. Kramer J. L. (2014). Cancer treatment and survivorship statistics. Ca. Cancer J. Clin. 64 (4), 252–271. 10.3322/caac.21235 24890451

[B19] Ding M. Duan X. Feng X. Wang P. Wang W. (2017). Application of CRS-PCR-RFLP to identify CYP1A1 gene polymorphism. J. Clin. Lab. Anal. 31 (6), e22149. 10.1002/jcla.22149 28213913 PMC6816910

[B20] El Mesallamy H. O. Rashed W. M. Hamdy N. M. Hamdy N. (2014). High-dose methotrexate in Egyptian pediatric acute lymphoblastic leukemia: The impact of ABCG2 C421A genetic polymorphism on plasma levels, what is next? J. Cancer Res. Clin. Oncol. 140 (8), 1359–1365. 10.1007/s00432-014-1670-y 24718721 PMC11823488

[B21] Erculj N. Kotnik B. F. Debeljak M. Jazbec J. Dolzan V. (2012). Influence of folate pathway polymorphisms on high-dose methotrexate-related toxicity and survival in childhood acute lymphoblastic leukemia. Leuk. Lymphoma 53 (6), 1096–1104. 10.3109/10428194.2011.639880 22074251

[B22] Esmaili M. A. Kazemi A. Faranoush M. Mellstedt H. Zaker F. Safa M. (2020). Polymorphisms within methotrexate pathway genes: Relationship between plasma methotrexate levels, toxicity experienced and outcome in pediatric acute lymphoblastic leukemia. Iran. J. Basic Med. Sci. 23 (6), 800–809. 10.22038/ijbms.2020.41754.9858 32695297 PMC7351433

[B23] Faganel Kotnik B. Grabnar I. Bohanec Grabar P. Dolzan V. Jazbec J. (2011). Association of genetic polymorphism in the folate metabolic pathway with methotrexate pharmacokinetics and toxicity in childhood acute lymphoblastic leukaemia and malignant lymphoma. Eur. J. Clin. Pharmacol. 67 (10), 993–1006. 10.1007/s00228-011-1046-z 21509569

[B24] Ferrari M. Guasti L. Maresca A. Mirabile M. Contini S. Grandi A. M. (2014). Association between statin-induced creatine kinase elevation and genetic polymorphisms in SLCO1B1, ABCB1 and ABCG2. Eur. J. Clin. Pharmacol. 70 (5), 539–547. 10.1007/s00228-014-1661-6 24595600

[B25] Forat-Yazdi M. Hosseini-Biouki F. Salehi J. Neamatzadeh H. Masoumi Dehshiri R. Sadri Z. (2016). Association between RFC1 G80A polymorphism and acute lymphoblastic leukemia: A review and meta-analysis of 10 studies. Iran. J. Ped. Hematol. Oncol. 6 (1), 52–63. 27222703 PMC4867172

[B26] Fotoohi K. Jansen G. Assaraf Y. G. Rothem L. Stark M. Kathmann I. (2004). Disparate mechanisms of antifolate resistance provoked by methotrexate and its metabolite 7-hydroxymethotrexate in leukemia cells: Implications for efficacy of methotrexate therapy. Blood 104 (13), 4194–4201. 10.1182/blood-2004-04-1493 15308564

[B27] Ghodke-Puranik Y. Puranik A. Shintre P. Joshi K. Patwardhan B. Lamba J. (2015). Folate metabolic pathway single nucleotide polymorphisms: A predictive pharmacogenetic marker of methotrexate response in Indian (asian) patients with rheumatoid arthritis. Pharmacogenomics 16 (18), 2019–2034. 10.2217/pgs.15.145 26616421 PMC4976849

[B28] Giletti A. Esperon P. (2018). Genetic markers in methotrexate treatments. Pharmacogenomics J. 18 (6), 689–703. 10.1038/s41397-018-0047-z 30237581

[B29] Gomez-Gomez Y. Organista-Nava J. Villanueva-Flores F. Estrada-Brito J. S. Rivera-Ramirez A. B. Saavedra-Herrera M. V. (2019). Association between the 5, 10-MTHFR 677C>T and RFC1 80G>A polymorphisms and acute lymphoblastic leukemia. Arch. Med. Res. 50 (4), 175–180. 10.1016/j.arcmed.2019.07.010 31499477

[B30] Greaves M. F. Wiemels J. (2003). Origins of chromosome translocations in childhood leukaemia. Nat. Rev. Cancer 3 (9), 639–649. 10.1038/nrc1164 12951583

[B31] Gregers J. Christensen I. J. Dalhoff K. Lausen B. Schroeder H. Rosthoej S. (2010). The association of reduced folate carrier 80G>A polymorphism to outcome in childhood acute lymphoblastic leukemia interacts with chromosome 21 copy number. Blood 115 (23), 4671–4677. 10.1182/blood-2010-01-256958 20335220 PMC2890175

[B32] Gutierrez-Camino A. Lopez-Lopez E. Martin-Guerrero I. Sanchez-Toledo J. Garcia de Andoin N. Carbone Baneres A. (2013). Intron 3 of the ARID5B gene: A hot spot for acute lymphoblastic leukemia susceptibility. J. Cancer Res. Clin. Oncol. 139 (11), 1879–1886. 10.1007/s00432-013-1512-3 24013273 PMC11824183

[B33] Han D. Shen C. Meng X. Bai J. Chen F. Yu Y. (2012). Methionine synthase reductase A66G polymorphism contributes to tumor susceptibility: Evidence from 35 case-control studies. Mol. Biol. Rep. 39 (2), 805–816. 10.1007/s11033-011-0802-6 21547363

[B34] Han F. Tan Y. Cui W. Dong L. Li W. (2013). Novel insights into etiologies of leukemia: A HuGE review and meta-analysis of CYP1A1 polymorphisms and leukemia risk. Am. J. Epidemiol. 178 (4), 493–507. 10.1093/aje/kwt016 23707957

[B35] Han J. Liu L. Meng L. Guo H. Zhang J. Han Z. Q. (2021). Effect of polymorphisms of ABCB1 and MTHFR on methotrexate-related toxicities in adults with hematological malignancies. Front. Oncol. 11, 759805. 10.3389/fonc.2021.759805 35004279 PMC8739189

[B36] Harmon D. L. Shields D. C. Woodside J. V. McMaster D. Yarnell J. W. Young I. S. (1999). Methionine synthase D919G polymorphism is a significant but modest determinant of circulating homocysteine concentrations. Genet. Epidemiol. 17 (4), 298–309. 10.1002/(SICI)1098-2272(199911)17:4<298:AID-GEPI5>3.0.CO;2-V 10520212

[B37] He H. R. Liu P. He G. H. Dong W. H. Wang M. Y. Dong Y. L. (2014). Association between reduced folate carrier G80A polymorphism and methotrexate toxicity in childhood acute lymphoblastic leukemia: A meta-analysis. Leuk. Lymphoma 55 (12), 2793–2800. 10.3109/10428194.2014.898761 24597986

[B38] Healy J. Richer C. Bourgey M. Kritikou E. A. Sinnett D. (2010). Replication analysis confirms the association of ARID5B with childhood B-cell acute lymphoblastic leukemia. Haematologica 95 (9), 1608–1611. 10.3324/haematol.2010.022459 20460642 PMC2930966

[B39] Holmboe L. Andersen A. M. Morkrid L. Slordal L. Hall K. S. (2012). High dose methotrexate chemotherapy: Pharmacokinetics, folate and toxicity in osteosarcoma patients. Br. J. Clin. Pharmacol. 73 (1), 106–114. 10.1111/j.1365-2125.2011.04054.x 21707700 PMC3248260

[B40] Howard S. C. McCormick J. Pui C. H. Buddington R. K. Harvey R. D. (2016). Preventing and managing toxicities of high-dose methotrexate. Oncologist 21 (12), 1471–1482. 10.1634/theoncologist.2015-0164 27496039 PMC5153332

[B41] Huang Z. Tong H. F. Li Y. Qian J. C. Wang J. X. Wang Z. (2016). Effect of the polymorphism of folylpolyglutamate synthetase on treatment of high-dose methotrexate in pediatric patients with acute lymphocytic leukemia. Med. Sci. Monit. 22, 4967–4973. 10.12659/msm.899021 27987364 PMC5189722

[B42] Jastaniah W. Elimam N. Abdalla K. AlAzmi A. A. Aseeri M. Felimban S. (2018). High-dose methotrexate vs. Capizzi methotrexate for the treatment of childhood T-cell acute lymphoblastic leukemia. Leuk. Res. Rep. 10, 44–51. 10.1016/j.lrr.2018.10.001 30416957 PMC6215054

[B43] Jekic B. Vejnovic D. Milic V. Maksimovic N. Damnjanovic T. Bunjevacki V. (2016). Association of 63/91 length polymorphism in the DHFR gene major promoter with toxicity of methotrexate in patients with rheumatoid arthritis. Pharmacogenomics 17 (15), 1687–1691. 10.2217/pgs-2016-0090 27636122

[B44] Jokic M. Brcic-Kostic K. Stefulj J. Catela Ivkovic T. Bozo L. Gamulin M. (2011). Association of MTHFR, MTR, MTRR, RFC1, and DHFR gene polymorphisms with susceptibility to sporadic colon cancer. DNA Cell Biol. 30 (10), 771–776. 10.1089/dna.2010.1189 21438757

[B45] Juliano R. L. Ling V. (1976). A surface glycoprotein modulating drug permeability in Chinese hamster ovary cell mutants. Biochim. Biophys. Acta 455 (1), 152–162. 10.1016/0005-2736(76)90160-7 990323

[B46] Kato T. Hamada A. Mori S. Saito H. (2012). Genetic polymorphisms in metabolic and cellular transport pathway of methotrexate impact clinical outcome of methotrexate monotherapy in Japanese patients with rheumatoid arthritis. Drug Metab. Pharmacokinet. 27 (2), 192–199. 10.2133/dmpk.dmpk-11-rg-066 22104130

[B47] Kotnik B. F. Jazbec J. Grabar P. B. Rodriguez-Antona C. Dolzan V. (2017). Association between SLC19A1 gene polymorphism and high dose methotrexate toxicity in childhood acute lymphoblastic leukaemia and non hodgkin malignant lymphoma: Introducing a haplotype based approach. Radiol. Oncol. 51 (4), 455–462. 10.1515/raon-2017-0040 29333125 PMC5765323

[B48] Kotur N. Lazic J. Ristivojevic B. Stankovic B. Gasic V. Dokmanovic L. (2020). Pharmacogenomic markers of methotrexate response in the consolidation phase of pediatric acute lymphoblastic leukemia treatment. Genes (Basel) 11 (4), E468. 10.3390/genes11040468 PMC7230684 32344632

[B49] Krajinovic M. (2008). MTHFD1 gene: Role in disease susceptibility and pharmacogenetics. Pharmacogenomics 9 (7), 829–832. 10.2217/14622416.9.7.829 18597647

[B50] Leclerc D. Wilson A. Dumas R. Gafuik C. Song D. Watkins D. (1998). Cloning and mapping of a cDNA for methionine synthase reductase, a flavoprotein defective in patients with homocystinuria. Proc. Natl. Acad. Sci. U. S. A. 95 (6), 3059–3064. 10.1073/pnas.95.6.3059 9501215 PMC19694

[B51] Li J. Wang X. R. Zhai X. W. Wang H. S. Qian X. W. Miao H. (2015). Association of SLCO1B1 gene polymorphisms with toxicity response of high dose methotrexate chemotherapy in childhood acute lymphoblastic leukemia. Int. J. Clin. Exp. Med. 8 (4), 6109–6113. 26131212 PMC4483916

[B52] Li S. Ye J. Liang E. Yang M. (2014). The protective role of MTR A2756G polymorphisms in childhood acute lymphoblastic leukemia remains inconclusive. Leuk. Lymphoma 55 (9), 2217–2218. 10.3109/10428194.2013.867490 24303783

[B53] Li W. X. Dai S. X. Zheng J. J. Liu J. Q. Huang J. F. (2015). Homocysteine metabolism gene polymorphisms (MTHFR C677T, MTHFR A1298C, MTR A2756G and MTRR A66G) jointly elevate the risk of folate deficiency. Nutrients 7 (8), 6670–6687. 10.3390/nu7085303 26266420 PMC4555142

[B54] Lincz L. F. Scorgie F. E. Garg M. B. Ackland S. P. (2007). Identification of a novel single nucleotide polymorphism in the first tandem repeat sequence of the thymidylate synthase 2R allele. Int. J. Cancer 120 (9), 1930–1934. 10.1002/ijc.22568 17278107

[B55] Liu S. Gao C. Zhang R. Jiao Y. Cui L. Li W. (2013). FPGS rs1544105 polymorphism is associated with treatment outcome in pediatric B-cell precursor acute lymphoblastic leukemia. Cancer Cell Int. 13 (1), 107. 10.1186/1475-2867-13-107 24168269 PMC3819686

[B56] Liu S. Gao C. Zhang R. Zhao X. Cui L. Li W. (2017). Polymorphisms in methotrexate transporters and their relationship to plasma methotrexate levels, toxicity of high-dose methotrexate, and outcome of pediatric acute lymphoblastic leukemia. Oncotarget 8 (23), 37761–37772. 10.18632/oncotarget.17781 28525903 PMC5514947

[B57] Lopez-Lopez E. Martin-Guerrero I. Ballesteros J. Garcia-Orad A. (2013). A systematic review and meta-analysis of MTHFR polymorphisms in methotrexate toxicity prediction in pediatric acute lymphoblastic leukemia. Pharmacogenomics J. 13 (6), 498–506. 10.1038/tpj.2012.44 23089671

[B58] Lopez-Lopez E. Martin-Guerrero I. Ballesteros J. Pinan M. A. Garcia-Miguel P. Navajas A. (2011). Polymorphisms of the SLCO1B1 gene predict methotrexate-related toxicity in childhood acute lymphoblastic leukemia. Pediatr. Blood Cancer 57 (4), 612–619. 10.1002/pbc.23074 21387541

[B59] Maksimovic V. Pavlovic-Popovic Z. Vukmirovic S. Cvejic J. Mooranian A. Al-Salami H. (2020). Molecular mechanism of action and pharmacokinetic properties of methotrexate. Mol. Biol. Rep. 47 (6), 4699–4708. 10.1007/s11033-020-05481-9 32415503

[B60] Mandal P. Samaddar S. Chandra J. Parakh N. Goel M. (2020). Adverse effects with intravenous methotrexate in children with acute lymphoblastic leukemia/lymphoma: A retrospective study. Indian J. Hematol. Blood Transfus. 36 (3), 498–504. 10.1007/s12288-019-01245-z 32647424 PMC7326748

[B61] Masson E. Relling M. Synold T. Liu Q. Schuetz J. Sandlund J. (1996). Accumulation of methotrexate polyglutamates in lymphoblasts is a determinant of antileukemic effects *in vivo*. A rationale for high-dose methotrexate. J. Clin. Invest. 97 (1), 73–80. 10.1172/JCI118409 8550853 PMC507064

[B62] Matsuo K. Hamajima N. Hirai T. Kato T. Inoue M. Takezaki T. (2002). Methionine synthase reductase gene A66G polymorphism is associated with risk of colorectal cancer. Asian pac. J. Cancer Prev. 3 (4), 353–359. 12716294

[B63] Mikkelsen T. S. Thorn C. F. Yang J. J. Ulrich C. M. French D. Zaza G. (2011). PharmGKB summary: Methotrexate pathway. Pharmacogenet. Genomics 21 (10), 679–686. 10.1097/FPC.0b013e328343dd93 21317831 PMC3139712

[B64] Moriyama T. Relling M. V. Yang J. J. (2015). Inherited genetic variation in childhood acute lymphoblastic leukemia. Blood 125 (26), 3988–3995. 10.1182/blood-2014-12-580001 25999454 PMC4481591

[B65] Muralidharan N. Misra D. P. Jain V. K. Negi V. S. (2017). Effect of thymidylate synthase (TYMS) gene polymorphisms with methotrexate treatment outcome in south Indian Tamil patients with rheumatoid arthritis. Clin. Rheumatol. 36 (6), 1253–1259. 10.1007/s10067-017-3608-7 28349270

[B66] Neradil J. Pavlasova G. Veselska R. (2012). New mechanisms for an old drug; DHFR- and non-DHFR-mediated effects of methotrexate in cancer cells. Klin. Onkol. 25, 2s87–92. 23581023

[B67] Niedzielska E. Węcławek-Tompol J. Matkowska-Kocjan A. Chybicka A. (2013). The influence of genetic RFC1, MS and MTHFR polymorphisms on the risk of acute lymphoblastic leukemia relapse in children and the adverse effects of methotrexate. Adv. Clin. Exp. Med. 22 (4), 579–584. official organ Wroclaw Medical University. 23986219

[B68] Niemi M. Pasanen M. K. Neuvonen P. J. (2011). Organic anion transporting polypeptide 1B1: A genetically polymorphic transporter of major importance for hepatic drug uptake. Pharmacol. Rev. 63 (1), 157–181. 10.1124/pr.110.002857 21245207

[B69] Nikbakht M. MalekZadeh K. Kumar Jha A. Askari M. Marwaha R. K. Kaul D. (2012). Polymorphisms of MTHFR and MTR genes are not related to susceptibility to childhood ALL in North India. Exp. Oncol. 34 (1), 43–48. 22453148

[B70] Ongaro A. De Mattei M. Della Porta M. Rigolin G. Ambrosio C. Di Raimondo F. (2009). Gene polymorphisms in folate metabolizing enzymes in adult acute lymphoblastic leukemia: Effects on methotrexate-related toxicity and survival. Haematologica 94 (10), 1391–1398. 10.3324/haematol.2009.008326 19648163 PMC2754955

[B71] Oosterom N. Berrevoets M. den Hoed M. A. H. Zolk O. Hoerning S. Pluijm S. M. F. (2018). The role of genetic polymorphisms in the thymidylate synthase (TYMS) gene in methotrexate-induced oral mucositis in children with acute lymphoblastic leukemia. Pharmacogenet. Genomics 28 (10), 223–229. 10.1097/FPC.0000000000000352 30222710

[B72] Park J. A. Shin H. Y. (2016). Influence of genetic polymorphisms in the folate pathway on toxicity after high-dose methotrexate treatment in pediatric osteosarcoma. Blood Res. 51 (1), 50–57. 10.5045/br.2016.51.1.50 27104192 PMC4828529

[B73] Pavlovic S. Kotur N. Stankovic B. Zukic B. Gasic V. Dokmanovic L. (2019). Pharmacogenomic and pharmacotranscriptomic profiling of childhood acute lymphoblastic leukemia: Paving the way to personalized treatment. Genes (Basel) 10 (3), E191. 10.3390/genes10030191 PMC6471971 30832275

[B74] Radtke S. Zolk O. Renner B. Paulides M. Zimmermann M. Moricke A. (2013). Germline genetic variations in methotrexate candidate genes are associated with pharmacokinetics, toxicity, and outcome in childhood acute lymphoblastic leukemia. Blood 121 (26), 5145–5153. 10.1182/blood-2013-01-480335 23652803

[B75] Ramsey L. B. Panetta J. C. Smith C. Yang W. Fan Y. Winick N. J. (2013). Genome-wide study of methotrexate clearance replicates SLCO1B1. Blood 121 (6), 898–904. 10.1182/blood-2012-08-452839 23233662 PMC3567337

[B76] Ramsey L. Bruun G. Yang W. Treviño L. Vattathil S. Scheet P. (2012). Rare versus common variants in pharmacogenetics: SLCO1B1 variation and methotrexate disposition. Genome Res. 22 (1), 1–8. 10.1101/gr.129668.111 22147369 PMC3246196

[B77] Robey R. To K. Polgar O. Dohse M. Fetsch P. Dean M. (2009). ABCG2: A perspective. Adv. Drug Deliv. Rev. 61 (1), 3–13. 10.1016/j.addr.2008.11.003 19135109 PMC3105088

[B78] Sajith M. Pawar A. Bafna V. Bartakke S. Subramanian K. Vaidya N. (2020). Serum methotrexate level and side effects of high dose methotrexate infusion in pediatric patients with acute lymphoblastic leukaemia (ALL). Indian J. Hematol. Blood Transfus. 36 (1), 51–58. 10.1007/s12288-019-01144-3 32174691 PMC7042471

[B79] Schmiegelow K. (2009). Advances in individual prediction of methotrexate toxicity: A review. Br. J. Haematol. 146 (5), 489–503. 10.1111/j.1365-2141.2009.07765.x 19538530

[B80] Schulte R. R. Choi L. Utreja N. Van Driest S. L. Stein C. M. Ho R. H. (2021). Effect of SLCO1B1 polymorphisms on high-dose methotrexate clearance in children and young adults with leukemia and lymphoblastic lymphoma. Clin. Transl. Sci. 14 (1), 343–353. 10.1111/cts.12879 32961024 PMC7877862

[B81] Shah P. P. Saurabh K. Pant M. C. Mathur N. Parmar D. (2009). Evidence for increased cytochrome P450 1A1 expression in blood lymphocytes of lung cancer patients. Mutat. Res. 670 (1-2), 74–78. 10.1016/j.mrfmmm.2009.07.006 19632247

[B82] Suthandiram S. Gan G. Zain S. Bee P. Lian L. Chang K. (2014). Effect of polymorphisms within methotrexate pathway genes on methotrexate toxicity and plasma levels in adults with hematological malignancies. Pharmacogenomics 15 (11), 1479–1494. 10.2217/pgs.14.97 25303299

[B83] Taylor Z. L. Vang J. Lopez-Lopez E. Oosterom N. Mikkelsen T. Ramsey L. B. (2021). Systematic review of pharmacogenetic factors that influence high-dose methotrexate pharmacokinetics in pediatric malignancies. Cancers (Basel) 13 (11), 2837. 10.3390/cancers13112837 34200242 PMC8201112

[B84] Tulstrup M. Moriyama T. Jiang C. Grosjean M. Nersting J. Abrahamsson J. (2020). Effects of germline DHFR and FPGS variants on methotrexate metabolism and relapse of leukemia. Blood 136 (10), 1161–1168. 10.1182/blood.2020005064 32391884 PMC7472715

[B85] Uhlén M. Fagerberg L. Hallström B. Lindskog C. Oksvold P. Mardinoglu A. (2015). Proteomics. Tissue-based map of the human proteome. Sci. (New York, N.Y.) 347 (6220), 1260419. 10.1126/science.1260419 25613900

[B86] van der Straaten R. Wessels J. de Vries-Bouwstra J. Goekoop-Ruiterman Y. Allaart C. Bogaartz J. (2007). Exploratory analysis of four polymorphisms in human GGH and FPGS genes and their effect in methotrexate-treated rheumatoid arthritis patients. Pharmacogenomics 8 (2), 141–150. 10.2217/14622416.8.2.141 17286537

[B87] van Schaik R. (2008). 6 dose adjustments based on pharmacogenetics of CYP450 enzymes. EJIFCC 19 (1), 42–47. 27683289 PMC4975340

[B88] Wang F. Liang Y. Wu X. Chen L. To K. Dai C. (2011). Prognostic value of the multidrug resistance transporter ABCG2 gene polymorphisms in Chinese patients with de novo acute leukaemia, Eur. J. Cancer, 47. 1990–1999. 10.1016/j.ejca.2011.03.032 21531129

[B89] Wang P. Deng Y. Yan X. Zhu J. Yin Y. Shu Y. (2020). The role of ARID5B in acute lymphoblastic leukemia and beyond. Front. Genet. 11, 598. 10.3389/fgene.2020.00598 32595701 PMC7303299

[B90] Wang S. M. Sun L. L. Zeng W. X. Wu W. S. Zhang G. L. (2014). Influence of genetic polymorphisms of FPGS, GGH, and MTHFR on serum methotrexate levels in Chinese children with acute lymphoblastic leukemia. Cancer Chemother. Pharmacol. 74 (2), 283–289. 10.1007/s00280-014-2507-8 24908438

[B91] Widemann B. C. Adamson P. C. (2006). Understanding and managing methotrexate nephrotoxicity. Oncologist 11 (6), 694–703. 10.1634/theoncologist.11-6-694 16794248

[B92] Wilson A. Platt R. Wu Q. Leclerc D. Christensen B. Yang H. (1999). A common variant in methionine synthase reductase combined with low cobalamin (vitamin B12) increases risk for spina bifida. Mol. Genet. Metab. 67 (4), 317–323. 10.1006/mgme.1999.2879 10444342

[B93] Wojtuszkiewicz A. Peters G. J. van Woerden N. L. Dubbelman B. Escherich G. Schmiegelow K. (2015). Methotrexate resistance in relation to treatment outcome in childhood acute lymphoblastic leukemia. J. Hematol. Oncol. 8, 61. 10.1186/s13045-015-0158-9 26022503 PMC4455979

[B94] Yang J. L. Liu Y. N. Bi Y. Y. Wang H. (2019). ARID5B gene polymorphisms and the risk of childhood acute lymphoblastic leukemia: A meta-analysis. Int. J. Hematol. 110 (3), 272–284. 10.1007/s12185-019-02658-2 31111395

[B95] Yang L. Wu H. Gelder T. V. Matic M. Ruan J. S. Han Y. (2017). SLCO1B1 rs4149056 genetic polymorphism predicting methotrexate toxicity in Chinese patients with non-Hodgkin lymphoma. Pharmacogenomics 18 (17), 1557–1562. 10.2217/pgs-2017-0110 29095107

[B96] Zaruma-Torres F. Lares-Asseff I. Reyes-Espinoza A. Loera-Castaneda V. Chairez-Hernandez I. Sosa-Macias M. (2015). Association of ABCB1, ABCC5 and xanthine oxidase genetic polymorphisms with methotrexate adverse reactions in Mexican pediatric patients with ALL. Drug Metab. Pers. Ther. 30 (3), 195–201. 10.1515/dmpt-2015-0011 26353179

[B97] Zhang H. Zhang Z. Li G. (2015). ABCB1 polymorphism and susceptibility to acute lymphoblastic leukemia: A meta analysis. Int. J. Clin. Exp. Med. 8 (5), 7585–7591. 26221303 PMC4509248

[B98] Zhang W. Sun S. Zhang W. Shi Z. (2018). Polymorphisms of ABCG2 and its impact on clinical relevance. Biochem. Biophys. Res. Commun. 503 (2), 408–413. 10.1016/j.bbrc.2018.06.157 29964015

[B99] Zhao M. Liang L. Ji L. Chen D. Zhang Y. Zhu Y. (2016). MTHFR gene polymorphisms and methotrexate toxicity in adult patients with hematological malignancies: A meta-analysis. Pharmacogenomics 17 (9), 1005–1017. 10.2217/pgs-2016-0004 27270164

[B100] Zhuo W. Zhang L. Qiu Z. Zhu B. Chen Z. (2012). Does cytochrome P450 1A1 MspI polymorphism increase acute lymphoblastic leukemia risk? Evidence from 2013 cases and 2903 controls. Gene 510 (1), 14–21. 10.1016/j.gene.2012.08.042 22964275

